# MDMX elevation by a novel Mdmx–p53 interaction inhibitor mitigates neuronal damage after ischemic stroke

**DOI:** 10.1038/s41598-022-25427-4

**Published:** 2022-12-06

**Authors:** Haomin Yan, Tsutomu Sasaki, Hideaki Kanki, Yoshiyuki Hirata, Kumiko Nishiyama, Sunao Hisada, Shigenobu Matsumura, Yasuo Nagaoka, Takaaki Sumiyoshi, Seiichi Nagano, Akiko Nakata, Minoru Yoshida, Shinichi Uesato, Hideki Mochizuki

**Affiliations:** 1 Department of Neurology, Graduate School of Medicine, Osaka University, Yamadaoka 2-2, Suita, Osaka 565-0871 Japan; 2 Department of Neurotherapeutics, Graduate School of Medicine, Osaka University, Yamadaoka 2-2, Suita, Osaka 565-0871 Japan; 3 Faculty of Pharmacy, Osaka Medical and Pharmaceutical University, 4-20-1 Nasahara, Takatsuki, Osaka 569-1094 Japan; 4 Hamamatsu Photonics, K.K. System Division, Joko-cho, Hamamatsu, Shizuoka 431-3196 Japan; 5 Graduate School of Comprehensive Rehabilitation, Osaka Prefecture University, Osaka, 583-8555 Japan; 6 Department of Life Science and Biotechnology, Faculty of Chemistry, Materials and Bioengineering, Kansai University, Yamate-cho 3-3-35, Suita, Osaka 564-8680 Japan; 7 Seed Compounds Exploratory Unit for Drug Discovery Platform, RIKEN Center for Sustainable Resource Science, 2-1 Hirosawa, Wako, Saitama 351-0198 Japan; 8 Chemical Genomics Research Group, RIKEN Center for Sustainable Resource Science, 2-1 Hirosawa, Wako, Saitama 351-0198 Japan

**Keywords:** Drug discovery, Neuroscience, Neurology

## Abstract

Mdmx and Mdm2 are two major suppressor factors for the tumor suppressor gene p53. In central nervous system, Mdmx suppresses the transcriptional activity of p53 and enhances the binding of Mdm2 to p53 for degradation. But Mdmx dynamics in cerebral infarction remained obscure. Here we investigated the role of Mdmx under ischemic conditions and evaluated the effects of our developed small-molecule Protein–Protein Interaction (PPI) inhibitors, K-181, on Mdmx–p53 interactions in vivo and in vitro. We found ischemic stroke decreased Mdmx expression with increased phosphorylation of Mdmx Serine 367, while Mdmx overexpression by AAV-Mdmx showed a neuroprotective effect on neurons. The PPI inhibitor, K-181 attenuated the neurological deficits by increasing Mdmx expression in post-stroke mice brain. Additionally, K-181 selectively inhibited HDAC6 activity and enhanced tubulin acetylation. Our findings clarified the dynamics of Mdmx in cerebral ischemia and provide a clue for the future pharmaceutic development of ischemic stroke.

## Introduction

Stroke is the leading cause of human death and long-term disability all over the world^1^. To date, although numerous clinical trials for stroke therapy have been accomplished, intravenous thrombolysis with recombinant tissue plasminogen activator (t-PA) remains the only clinical effective treatment^2^. However, the narrow therapeutic window and the rapid progression of brain injury following ischemia restrict its clinical application^3^.

The tumor suppressor gene p53 plays an important role in the development of the CNS^4,5^. In addition, p53-dependent neuronal apoptosis is associated with neuronal damage in both acute injury such as stroke and chronic neurodegenerative diseases^6–8^. p53 contributes not only to apoptosis but also to necrosis by controlling the interaction of cyclophilin D with permeability transition pores in mitochondria^9^. Accumulated research has shown that p53 plays a central role in neuronal cell death after cerebral infarction, and its suppression reduces ischemic cell damage^7,10–15^.

The two major suppressors of p53 are Mouse Double Minute 2 (Mdm2) and Mdmx (also called Mdm4)^16^. Mdm2 is the predominant E3 ligase for p53 and degrades p53 by promoting ubiquitination of p53. On the other hand, Mdmx suppresses the transcriptional activity of p53 and enhances the binding of Mdm2 to p53 for degradation. The role of the Mdm2–p53 pathway after cerebral ischemia has already been reported^17,18^. Mdmx, a homologue of E3 ligase Mdm2, has been mainly studied as a p53-dependent cancer regulatory protein so far^19,20^, and most of the existing data are obtained from cancer studies^21^. Recent studies suggested an important role of Mdmx in normal neuron development. Mdmx modifications like Ring deletion^22^ and C462A knock in^23^ to alter its Mdm2 binding ability leaded to mice embryonic lethal. General Mdmx knock-out mice died at embryonic day 7.5–9.5^24^, and conditional Mdmx deletion in CNS using a Nestin-Cre mouse model resulted in lethal deficits at embryonic day 17.5^25^. Moreover, accumulative studies suggest an irreplaceable role of Mdmx not only in normal neuron development but also in various CNS diseases and therapies^26,27^. Neuronal stress signals induce Mdmx loss, whereas Mdmx knockdown favored neuronal apoptosis, suggesting Mdmx contributes to neurons survival in response to stresses^28^. In addition, Mdmx overexpression attenuates HIV-induced neurotoxicity^29^. In aggregate, these findings underscore a critical role of Mdmx in normal CNS development and maintenance.

To the date, most of the pharmacological approaches aiming at reactivating p53 in cancer cells have focused on the interaction interface between p53 and Mdm2 or Mdmx^30^, and most protein–protein interaction (PPI) inhibitors are peptides or antibody drugs. We previously reported new low-molecular-weight Mdmx–p53 disruptors and their anti-cancer activities^31^. Among them, *S-*2-benzamidophenyl 2-methylpropanethioate (K-181) and its free thiol derivative (K-181SH) preferentially inhibited Mdmx–p53 interaction over Mdm2–p53 interaction and suppressed the growth of cancer cells. We speculate that K-181, after being hydrolyzed to K-181SH in the body, interfered with the p53–Mdmx interaction through disulfide-bond formation between the free thiol groups of K-181SH and Cys77 in Mdmx in the p53-binding domain. Here, we investigated the effects of K-181 on ischemic stroke both in vitro and in vivo, and found that K-181 and its free thiol derivative K-181SH induced Mdmx up-regulation. More interestingly, K-181 and K-181SH showed an HDAC6 inhibitory effect. HDAC6 is a cytoplasmic Class II histone deacetylase and has a deacetylase activity on several cytoskeleton proteins including tubulin^32^. Dysregulated HDAC6 restores tubulin acetylation^33^, and correlates against neuronal microtubule instability^34^, cognition impairment^35^ and peripheral neuropathy development^36^, while HDAC6 inhibition attenuates neuronal damages under cell stresses^37^. Therefore, selectively targeting HDAC6 may alleviate neuropathic damages^38^.

Despite countless efforts been made for development of drugs that inhibit the interaction of p53 with Mdm2 or Mdmx in cancers, the role of Mdmx after cerebral ischemia and its possibility as a therapeutic drug have not yet been reported. In this study, we clarified the dynamics of Mdmx after cerebral ischemia, and demonstrated the neuroprotective effects of our newly developed small-molecule PPI inhibitors by modifying the interactions between Mdmx and p53 and HDAC6 activity inhibition.

## Materials and methods

Details for all methods are provided in the Supplemental Material.

### Animals

8-week-old male C57BL/6J male mice were purchased from Charles River Japan, Inc. and raised under standard conditions of light (lights on: 8:00 a.m.–8:00 p.m.) and temperature (23 °C, 40% humidity). A totally of 130 mice were included in this study. All experimental procedures followed the guidelines and were fully approved by the Ethics Committee for Animal Experiments of Osaka University Graduate School of Medicine. All experiments were conducted in compliance with the Animal Research: Reporting of In Vivo Experiments (ARRIVE) guidelines.

### Drug synthesis

Small-molecule PPI inhibitors, K-181, K-181SH, K-178 (*S-*2-Isobutyramidophenyl 2-methylpropanethioate) and K-178SH, were synthesized according to the reported procedures^31^.

### Inhibition assay

1. **Measurement of the inhibition of the small-molecule PPI inhibitors, K-181, K-181SH, K-178 and K-178SH using the modified ELISA**.
  Inhibitory activities (IC_50_s) of the PPI inhibitors against the FLAG-p53 and GST–protein interaction measured by the modified ELISA were previously reported^31^.
2. **HDAC inhibitory activity assay of the PPI inhibitors**.
  Inhibitory activities of the PPI inhibitors on HDACs were measured utilizing a fluorogenic assay as described in the reports^39,40^.

### Recombinant AAV9 vectors

Mice Mdm2 and Mdmx were cloned in our lab and inserted into pAAV-MCS Expression vector (CELL BIOLABS INC, San Diego, CA) using flanking BamHI and XhoI restriction sites. Briefly, AAV 293 were transfected with rAAV (pUCmini-iCAP-PHP.eB (addgene plasmid #103005, Watertown, MA) and rAAV-ITR Mdmx or Mdm2 expression plasmid (single stranded genome) or pAAV-GFP Control Plasmid (CELL BIOLABS INC, San Diego, CA) and helper plasmid (Agilent Technologies, Santa Clara, CA) by the calcium phosphate method. 120 h after transfection cells were harvested and vector purified using a standard iodixanol density gradient and ultracentrifugation protocol. Iodixanol was removed and vector concentrated in PBS by diafiltration using Amicon Ultra 100 kDa MWCO centrifugal devices (Millipore, Billerica, MA). Vector was stored at − 80 °C until use. rAAV titers were determined by quantitative PCR and expressed as genome copies per ml (gc/ml). They AAV particles were applied to the primary neuronal culture at MOI of 5 × 10^4^ at 3 days in vitro and incubated for 3 days.

### Lentivirus production

Lentivirus production was performed as previously described^41^. Construct for lentiviral vector (pLKO.1) expressing control shRNA (SHP002) and a shRNA against mouse p53 gene (Trp53) (Cat. TRCN0000173949) were purchased from Sigma-Aldrich. Briefly, 293FT cells were transfected with pCAG-HIVgp (RIKEN BioResource Center, #RDB04394), pCMV-VSV-G-RSV-Rev (RIKEN BioResource Center, #RDB04393) and a shRNA construct using the CalPhos™ Mammalian Transfection Kit (Takara). pCAG-HIVgp and pCMV-VSV-G-RSV-Rev were developed by the late Dr. Hiroyuki Miyoshi and provided by the RIKEN BRC through the National BioResource Project of the MEXT, Japan^42^. The lentivirus-containing medium was collected at 48 h and 72 h after transfection and ultracentrifuged at 70,000*g* for 140 min. The pellet was resuspended and applied for primary neuronal transduction at MOI of 10 at 5 days in vitro and incubated for 4 days.

### Transient middle cerebral artery occlusion

Transient Middle Cerebral Artery Occlusion (tMCAO) was conducted as previously described^43^. Mice were randomly distributed into K-181 and vehicle treated groups. General anesthesia was rapid conducted with 3% isoflurane and maintained at 1.5% via an open mask. The right middle cerebral artery was occluded for 60 min with a suture. The reperfusion of mice was verified by both laser-Doppler flowmetry (Advanced Laser Flowmetry) and a 2D laser blood flow imager (omegazone, OZ-1). Laser-Doppler flowmetry was used to monitor cortical cerebral blood flow (CBF) by attachment to the exposure skull from pre until 15 min after operation, and mice with more than 30% of baseline during the first minute of occlusion were excluded. After the operation, mice were returned to their individual cages. During the whole surgery, the body temperature was monitored by a rectal probe and maintained at 37.0 ± 0.5 °C.

### Behavior test

After 48 h of recovery, foot fault test and neurological score were performed to evaluate mice neurological deficits. For the foot fault test, mice were placed on an elevated gridded platform above the surface and allowed to walk for 5 min. A foot fault was noted when the left forefoot misstepped and fell through the space between the grids. The percentage of left foot faults was measured for statistical analysis during the whole observation. For neurological score, a criteria described in a previous study^44^ was used. Spontaneous activity, symmetry of movements, symmetry of forelimbs, climbing wall of wire cage, reaction to touch on either side of trunk and response to vibrissae touch were scored as 0 to 3 points respectively. Lower score indicates worse neurological deficits. Neurological tests were evaluated by 2 researchers independently.

### CBF measurement

The measurement of CBF was conducted 24 h after tMCAO as previously described^45^. Surface CBF was recorded by a laser speckle blood flow imaging system (Omegazone OZ-1). After general anesthesia, the skull was exposed by a midline scalp incision. The surface of the skull was wiped clean with saline-soaked gauze before recording. Color-coded CBF images were obtained in high-resolution mode.

### Quantitative real-time PCR analysis

After the total RNA extraction of brains and cultures using mirVana™ miRNA Isolation kit (Thermo Fisher Scientific), cDNA was prepared from 1 μg total RNA using the SuperScript VILO cDNA Synthesis Kit (Invitrogen). Power SYBR Green PCR Master Mix (Thermo Fisher Scientific) was used for real-time PCR. 36B4 served as endogenous control. Relative expression was calculated using the ΔΔCt method with the QuantStudio 7 Real Time PCR System (Applied Biosystems). Primer sequences were described as below: p53: F: 5ʹ-GTATTTCACCCTCAAGATCC-3ʹ, R: 5ʹ-TGGGCATCCTTTAACTCTA-3ʹ. Mdmx: F: 5'-CCATCTGACGACATGTTTCC-3ʹ, R: 5'-TTACAAGCAGGACACGAAGC-3ʹ. 36B4: F: 5ʹ-TGTGTGTCTG CAGATCGGGT-3ʹ, R: 5ʹ-TGGATCAGCCAGGAAGGCCT-3ʹ.

### Statistical analysis

Comparisons between two separate groups were performed using unpaired non-parametric *t* test. Multiple groups comparisons were performed using one-way ANOVA with Bonferroni’s post hoc test. All data are presented as means ± standard deviation (SD).

## Results

### Mdmx exerts a neuroprotective role in neuron

**Figure 1:**
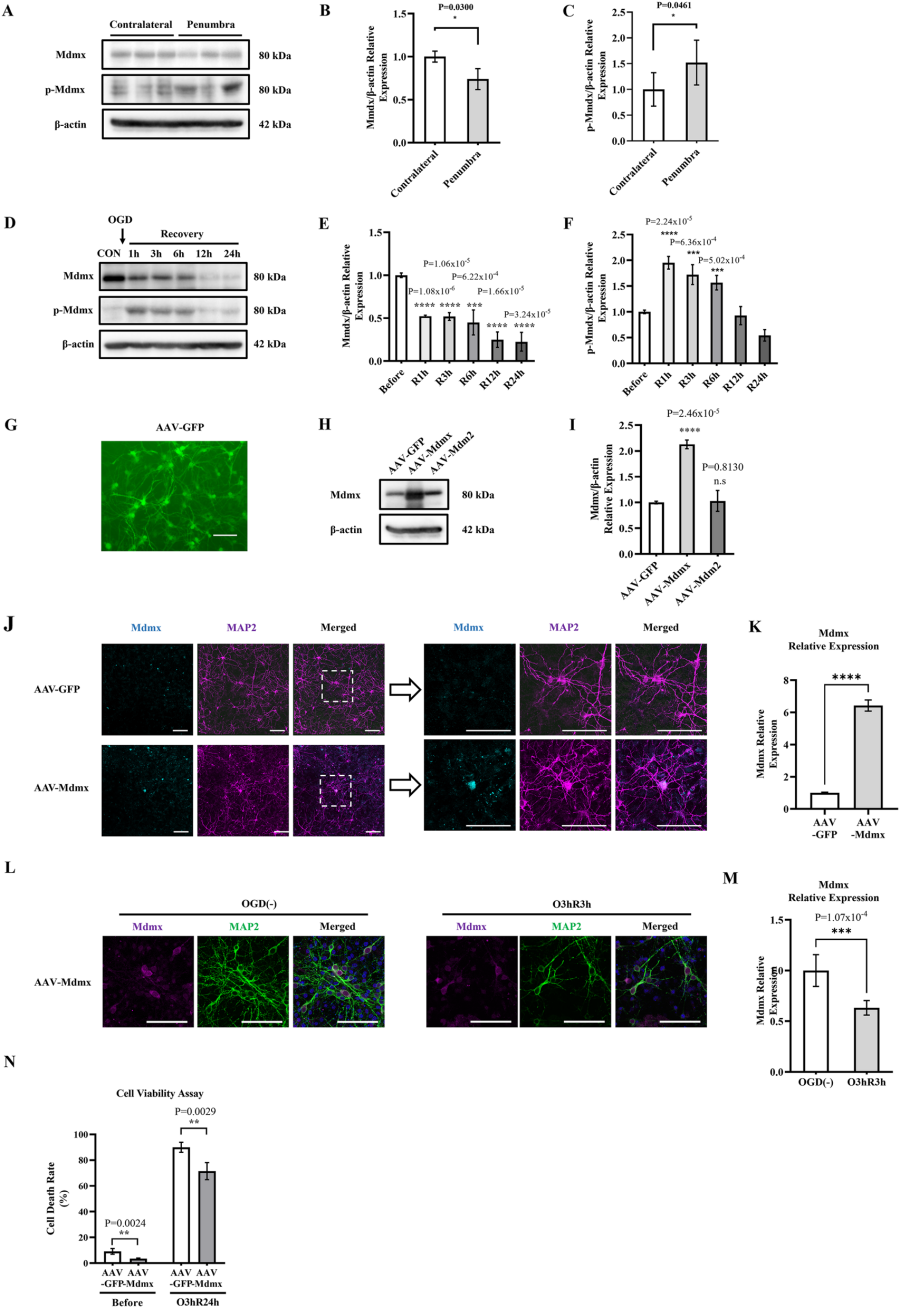
Mdmx exerts a neuroprotective role in hypoxia/recovery. (**A**–**C**) Western blot (**A**) and quantification of Mdmx (**B**) and p-Mdmx (**C**) in mice contralateral and penumbra. (**D**–**F**) Western blot (**D**) and quantification of Mdmx (**E**) and p-Mdmx (**F**) in neurons, before and after hypoxia/recovery. N = 3. (**G**) Successful cell transduction is demonstrated by expression of AAV-GFP. Scale Bar = 100 μm. (**H**,**I**) Western blot (**H**) and quantification of Mdmx (**I**) in AAV-GFP, AAV-Mdmx and AAV-Mdm2 transduced neurons. N = 3. (**J**,**K**) Immunofluorescence Stanning (**J**) and quantification (**K**) of Mdmx/MAP2 in AAV-GFP and AAV-Mdmx transduced neurons. N = 6. Scale Bar = 100 μm. (**L**,**M**) Immunofluorescence Stanning (**L**) and quantification (**M**) of Mdmx/MAP2 in AAV-Mdmx transduced neurons, before and after OGD. N = 6. Scale Bar = 100 μm. (**N**) Cell Viability Assay of AAV-GFP and AAV-Mdmx transduced neurons before and after hypoxia/recovery. N = 4.

To investigate the role of Mdmx in stroke, we applied C57BL/6J wild-type mice to 60 min tMCAO, then investigated the Mdmx expression in mice brain (Fig. 1A). Total Mdmx expression significantly decreased with increasing phosphorylated Mdmx in the penumbra area 24 h after tMCAO (Fig. 1B,C). To clarify the Mdmx expression in neurons, we subjected primary neurons to OGD for 3 h following reperfusion up to 24 h (Fig. 1D). Decreased Mdmx and elevated p-Mdmx were observed after hypoxia up to 6 h after OGD (Fig. 1E,F). In order to investigate the role of Mdmx in neuron survival, we cloned and produced recombinant adeno-associated virus (rAAV) which expressed GFP, Mdm2, and Mdmx, respectively. On day in vitro 4, cells demonstrated extensive neuronal processes and were well differentiated. GFP transgene expression was observed in infected neuronal cultures 72 h after transfection, indicating successful transduction (Fig. 1G). AAV-Mdmx transduction significantly increased Mdmx expression compared to AAV-GFP, while AAV-Mdm2 barely altered Mdmx expression (Fig. 1H,I). Confocal microscopy showed AAV-Mdmx transduction-conducted Mdmx expression is mainly localized in the soma rather than axons of neurons (Fig. 1J,K). Subsequently, we subjected neurons transduced with AAV-GFP/AAV-Mdmx to Oxygen Glucose Deprivation (OGD) following 3 h and 24 h reperfusion. Mdmx expression decreased significantly in response to OGD (Fig. 1L,M), and AAV-Mdmx transduced neurons showed less cell death than AAV-GFP transduced neurons before and after OGD, inferring Mdmx overexpression reduced ischemic vulnerability hypoxia/recovery (Fig. 1N). Furthermore, to assess whether AAV-Mdmx-induced Mdmx expression affects p53 activity, we conducted western blot using neuronal cultures transfected with AAV-GFP and AAV-Mdmx, respectively. We found that compared to neurons transfected with AAV-GFP, p53 protein expression was not altered in AAV-Mdmx transfected neurons (Supplemental Fig. 1). Further studies are needed to investigate whether Mdmx affects p53 transcript activity. These results indicate Mdmx exerts a crucial role in neurons.

### K-181 inhibits Mdmx–p53 interaction more than Mdm2–p53 interaction and increased Mdmx expression in mice brain under physiological condition

**Figure 2:**
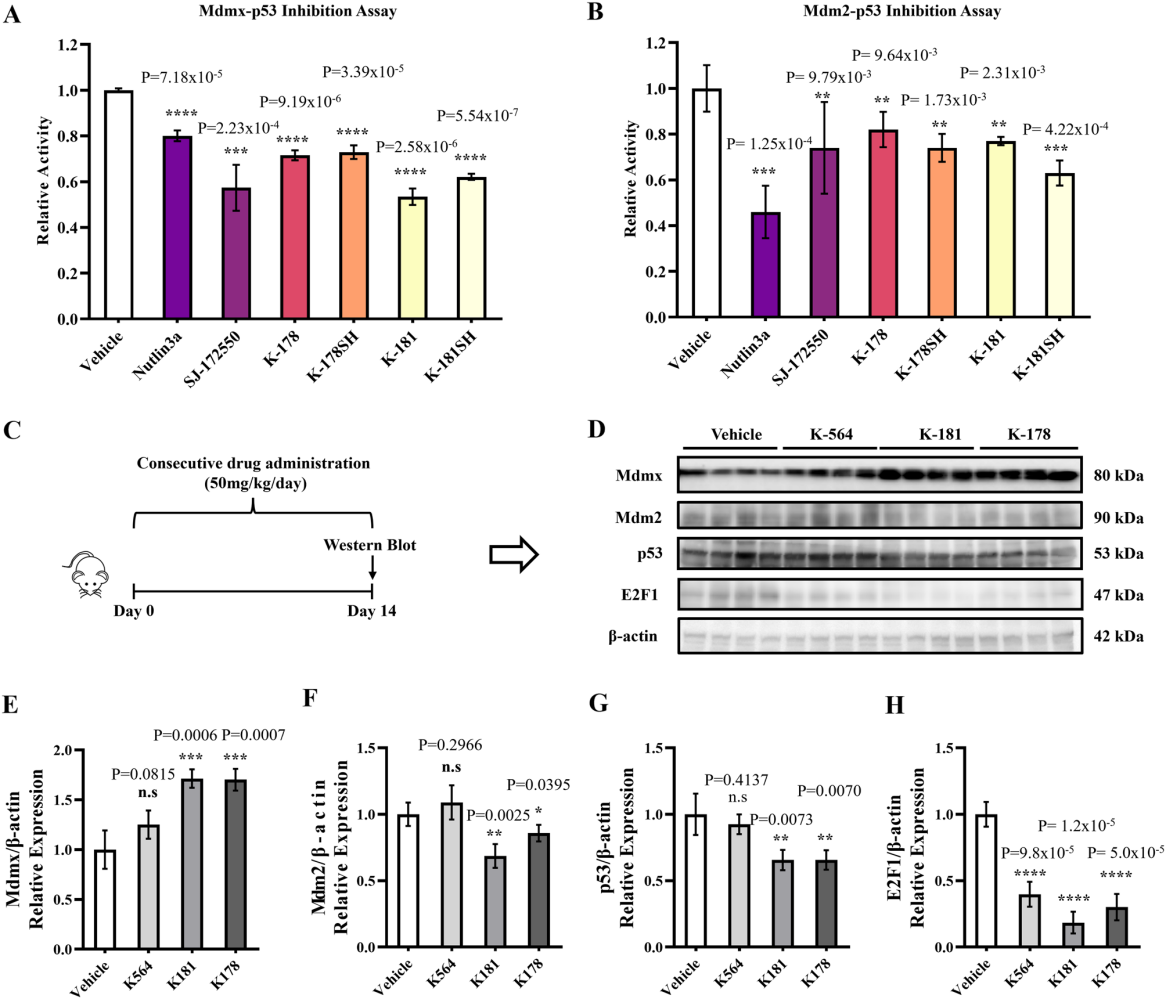
K-181 increased Mdmx expression in mice brain under physiological condition. (**A**,**B**) Relative inhibition activity on Mdmx–p53 (**A**) and Mdm2–p53 (**B**) interaction using chemical compounds at 12.5 μM. N = 3. (**C**) Mice were orally administrated with K-181, K-178, K-564 and vehicles at 50 mg/kg/day for consecutive 14 days. (**D**–**H**) Western blot (**D**) and quantification of Mdmx (**E**), Mdm2 (**F**), p53 (**G**) and E2F1 (**H**) in mice brains. N = 4.

The neuroprotective effect of elevated Mdmx on neuronal survival inspired us to search compounds that increase Mdmx from our own PPI library^32^ including Mdm2/Mdmx–p53 inhibitors, since Mdm2/Mdmx–p53 pathway could be linked to alteration of Mdmx expression. Firstly, we conducted inhibition assay of the small-molecule PPI inhibitors against Mdmx–p53 and Mdm2–p53 interaction (Fig. 2A,B). SJ-172550^46^ was used for positive control as a well-studied Mdmx–p53 inhibitor. Compared to the control group, K-181 and its free thiol derivative K-181SH showed significant inhibitory effects on Mdmx–p53 interaction, as previously reported. To elucidate the effects of these small-molecule inhibitors on p53–Mmdx interaction in vivo, we subjected C57BL/6J male mice to consecutive 14 days oral administration of K-181, K-178 and K-564 (Fig. 2C) to examine Mdmx expression in brain (Fig. 2D). The HDAC inhibitor K-564 which has been previously reported by us as a neuroprotective drug^47^ is used here as a chemical control since it is a HDAC inhibitor but does not act on Mdmx–p53 interaction. After 14 days of oral administration, K-181- and K-178-treated mice showed a statistically elevated Mdmx (Fig. 2E) while a slightly decreased Mdm2 (Fig. 2F) and p53 (Fig. 2G) expression in brain, compared with vehicle treated mice.

Extensive evidence showed that both p53 and E2F1 are implicated in neuronal apoptosis, and correlate with Mdmx in various cancer cells^48^. Furthermore, a decrease in E2F1 expression was observed in mice treated with K-181 and K-178 (Fig. 2H). These data indicate that K-181 administration elevates Mdmx and decreases p53 and Mdm2 protein expression in vivo. Since K-181 displayed the most obvious influence on alteration of Mdmx, P53 and E2F1 expression, we focused on K-181 effects upon ischemic stroke in the subsequent experiments.

### K-181 attenuates acute ischemic stroke damage in mice

**Figure 3:**
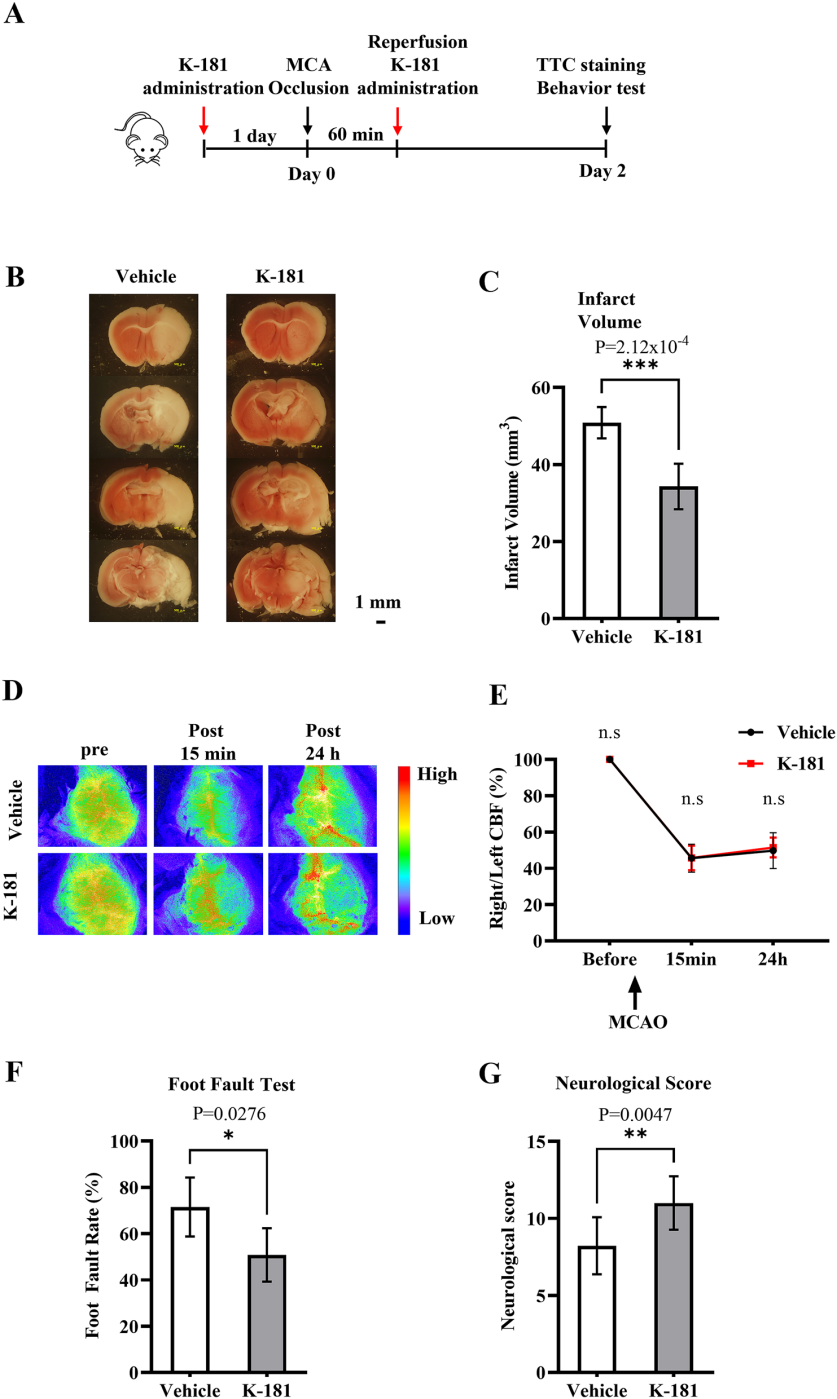
K-181 attenuates acute ischemic stroke damage in mice. (**A**) Mice were orally administrated with K-181 (50 mg/kg) twice, 1 day before and immediately after tMCAO. (**B**,**C**) Representative TTC staining images (**B**) and quantification (**C**) of mice infarct size 2 days after tMCAO. N = 6. Scale Bar = 1 mm. (**D**,**E**) Representative images (**D**) and quantification (**E**) of mice cerebral blood flow visualized by Omegazone. N = 6. (**F**) Foot Faut test of mice 48 h after tMCAO. N = 5. (**G**) Neurological score of mice 48 h after tMCAO. N = 9.

To investigate K-181 effects on ischemic stroke, we conducted tMCAO on C57BL/6J male mice. K-181 was orally administrated twice 1 day before and immediately after tMCAO (Fig. 3A), and we found K-181 significantly reduced the infarct volume 2 day after tMCAO (Fig. 3B,C) compared to the vehicle treated mice. No differences of cerebral blood flow (CBF) were found up to 15 min and 24 h after tMCAO between K-181 and vehicle groups (Fig. 3D,E). Furthermore, foot fault test and neurological score were performed to evaluate mice neurological deficits 48 h after tMCAO. K-181 administration attenuated the worsened forelimb use in the foot fault test (Fig. 3F) and the neurological score (Fig. 3G) 48 h after tMCAO. These data indicate that K-181 exerts a neuroprotective effect on ischemic stroke.

### K-181 increases Mdmx expression under ischemic stroke in both in vivo and in vitro

**Figure 4:**
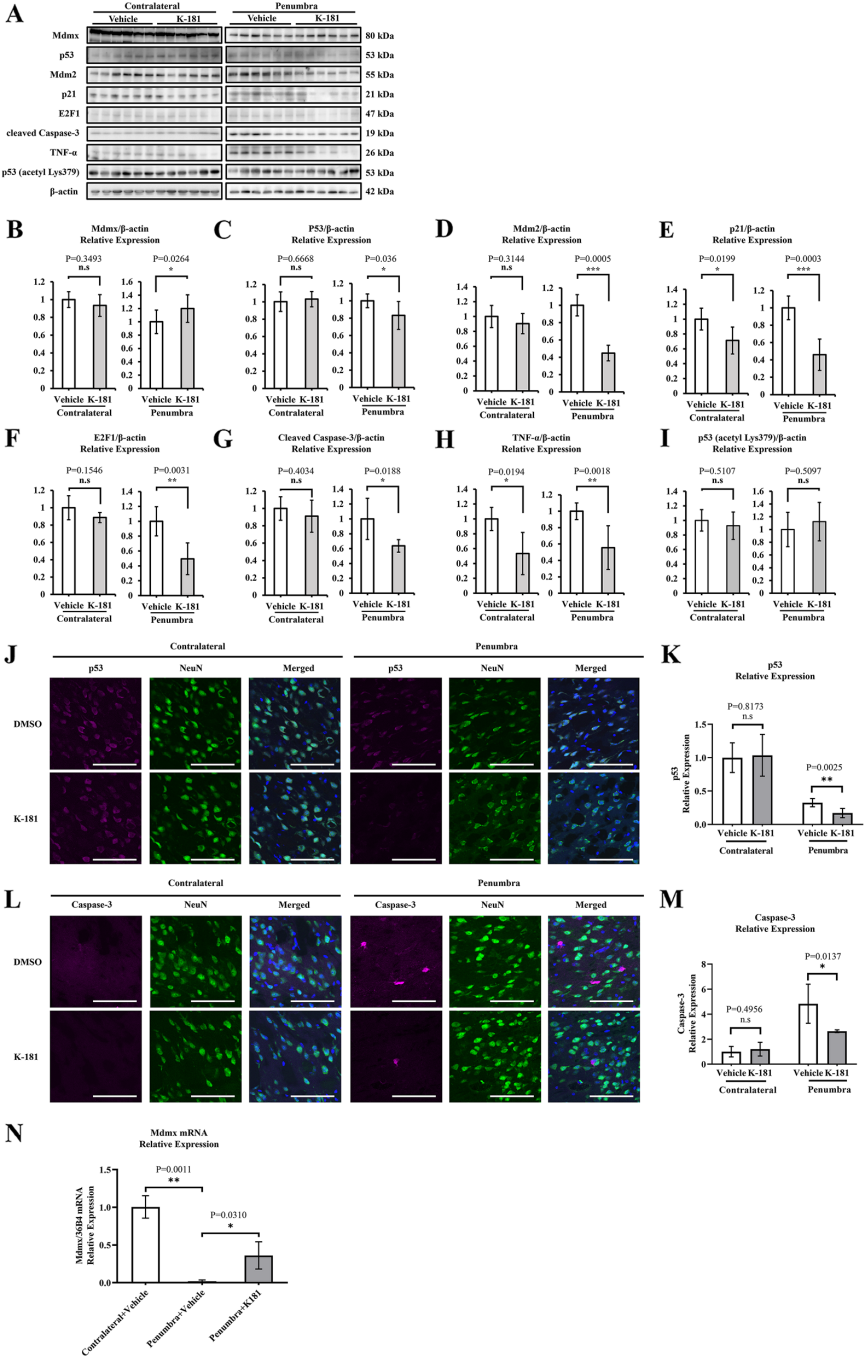
K-181 increases Mdmx expression under ischemic stroke in mice brains. (**A**–**I**) Western Blot images (**A**) and quantification of Mdmx (**B**), p53 (**C**), Mdm2 (**D**), p21 (**E**), E2F1 (**F**), cleaved Caspase-3 (**G**), TNF-α (**H**) and p53 (acetyl Lys379) (**I**) expression in vehicle and K-181 treated mice brains. (**J**–**K**) Representative immunostaining images (**J**) and quantification of p53 (**K**) in vehicle and K-181 treated mice brains. N = 6. Scale Bar = 100 μm. (**L**–**M**) Representative immunostaining images (**L**) and quantification of Caspase-3 (**M**) in vehicle and K-181 treated mice brains. N = 6. Scale Bar = 100 μm. (**N**) qPCR results of Mdmx mRNA levels in vehicle and K-181 treated mice brains. N = 3.

We investigated the mechanism of neuroprotective effects of K-181 oral administration (Fig. 4A). Under ischemic condition, K-181 administration elevated Mdmx expression (Fig. 4B) and decreased p53 (Fig. 4C), Mdm2 (Fig. 4D), p21 (Fig. 4E) and E2F1 (Fig. 4F) expression in penumbra regions. Additionally, cleaved Caspase-3 (Fig. 4G) and TNF-α (Fig. 4H) expression were significantly decreased in K-181 treated mice, inferring a neuroprotective effect of K-181. We also evaluated the effects of K-181 on p53 acetylation, and found K-181 administration did not alter acetylated p53 expression (Fig. 4I). To visualize the effects of K-181 on p53 localization, we conducted immunofluorescence co-staining of p53/NeuN in brain sections. We found that K-181 administration enhanced the decrease of p53 induced by ischemic stroke (Fig. 4J,K). Next, we investigated the role of K-181 on acetylation of p53 using immunofluorescence co-staining of p53 (acetyl Lys379) and NeuN. We found that K-181 did not alter the acetylation of p53 before and after ischemia/reperfusion (Supplemental Fig. 2), consistently with the western blot results. We also conducted immunofluorescence co-staining of Caspase-3/NeuN in brain sections, and observed that K-181 decreased the induction of Caspase-3 by ischemic stroke (Fig. 4L,M). Furthermore, to clarify whether transcription is affected, we evaluated the mRNA levels of Mdmx in mice brains by qPCR. Consistently, we found that K-181 attenuated the decrease of Mdmx mRNA induced by ischemic stroke (Fig. 4N), suggesting that K-181 increases MDMX levels, at least in part, through transcriptional activation in the postischemic brain. We also detected the mRNA of p53 in mice brain to discuss the effect of K-181 on p53 level and transcription activity, and we found that K-181 decreased the p53 mRNA level in mice penumbra (Supplemental Fig. 3), consistently with the western blot data.

**Figure 5:**
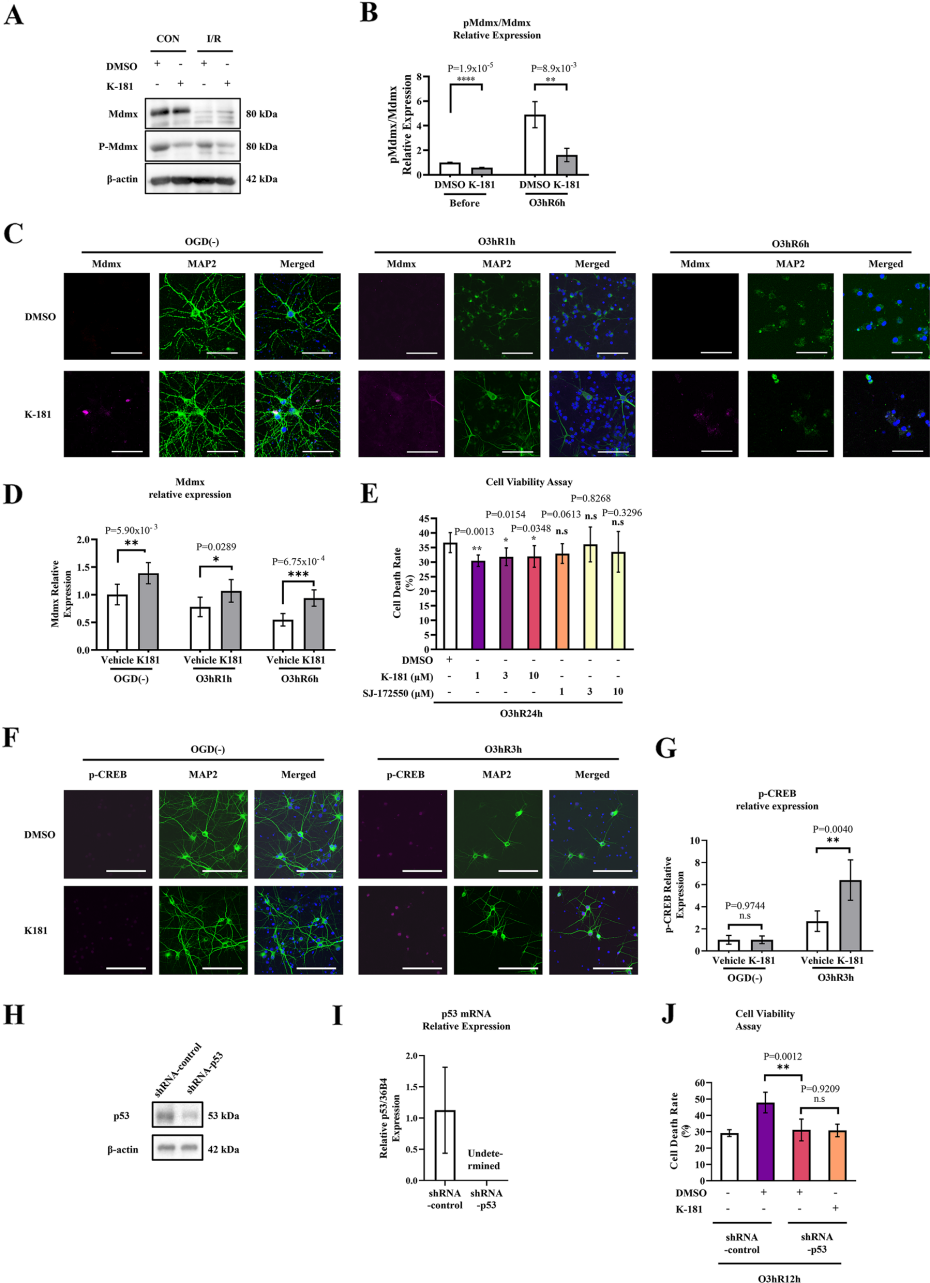
K-181 exerts neuroprotective effects in response to hypoxia/reperfusion in neurons. (**A**,**B**) Representative western blot images (**A**) and quantification of pMdmx/Mdmx relative expression (**B**) in neurons which were pre-treated with DMSO and 10 μM of K-181 after 3 h OGD following 9 h reperfusion. N = 3. (**C**,**D**) Representative immunostaining images (**C**) and quantification (**D**) of Mdmx in neurons, before and after hypoxia/recovery. N = 6. Scale Bar = 100 μm. (**E**) Cell death rate of neurons which were pre-treated with DMSO, K-181 and SJ-172550 at 24 h after 3 h OGD. N = 4–6. (**F**,**G**) Representative immunostaining images (**F**) and quantification (**G**) of phosphorylated cyclic AMP-responsive element-binding protein (p-CREB) in neurons, before and after hypoxia/recovery. N = 6. Scale Bar = 100 μm. (**H**) Western blot of p53 in neurons transduced with shRNA-control and shRNA-p53. (**I**) qPCR of p53 mRNA in neurons transduced with shRNA-control and shRNA-p53. N = 3. (**J**) Cell death rate of neurons which were transfected with shRNA-control and shRNA-p53 at 12 h after 3 h OGD. N = 4–6.

To clarify the effect of K-181 on neurons in response to ischemia/reperfusion, we added K-181 to primary neuronal cultures, and subjected these cultures to 3 h OGD, following 6 h reperfusion (Fig. 5A). We found that compared to DMSO group, K-181 treated neurons showed a decreased p-Mdmx/Mdmx expression (Fig. 5B), especially after hypoxia/reperfusion. To evaluate the regulatory role of K-181 on Mdmx in neuron, we treated primary neuronal cultures with 10 μM K-181 and DMSO, respectively, and we found K-181 administration increased Mdmx expression in neurons. After subjection to 3 h OGD, both K-181 and DMSO treated neurons showed a decrease of Mdmx expression, while K-181 attenuated Mdmx decrease (Fig. 5C,D). We subjected both K-181 and DMSO treated neurons to 3.5 h OGD, following 24 h reperfusion. SJ-172550, a well-studied Mdmx–p53 inhibitor, was used here for positive control. We found that compared to DMSO group, the death rate of K-181 treated neuron after OGD significantly declined (Fig. 5E). SJ-172550 showed little neuroprotection against OGD/reperfusion, which may because the limited drug concentration applied. Trial using different concentration are needed to elucidate the neuroprotective effects of SJ-172550. Moreover, we evaluated Annexin V/propidium iodide (PI) translocation in neurons before and after OGD. We found that DMSO induced Annexin V and PI translocation before and after OGD, while K-181 decreased PI translocation (Supplemental Fig. 4). To furtherly analyze cell viability and functional activation in neurons, we performed immunofluorescence co-staining of phosphorylated cyclic AMP-responsive element-binding protein (p-CREB) and MAP2 in neurons. CREB was phosphorylated in response to OGD/Reperfusion, while K-181 enhanced the phosphorylation (Fig. 5F,G), indicating an attenuated cell death^49^. In addition, to investigate whether Mdmx-mediated neuroprotection is dependent on p53, we constructed lentivirus to knock down p53 in vitro and evaluate the effect of p53 deficiency on neuron vulnerability to ischemia/reperfusion with or without K-181 treatment. P53 was successfully knocked down and confirmed by both western blot (Fig. 5H) and qPCR (Fig. 5I). Next, LDH assay was used to evaluate p53 knock-down and control neuron vulnerability to ischemia/reperfusion after treatment of K-181 or DMSO, and we found that p53 knock down significantly decreased neuron death after I/R, while K-181, which inhibits p53 and elevates Mdmx expression, showed little protection on p53 knock-down neuron (Fig. 5J), suggesting the Mdmx-mediated neuroprotection induced by K-181 is mainly dependent on p53. Taken together, these data above suggest a neuroprotective role of K-181 in ischemic stroke via enhancing Mdmx expression, and further studies are needed to elucidate the role of Mdmx on neuroprotection.

### K-181 selectively inhibits HDAC6 activity and enhances tubulin acetylation

**Figure 6:**
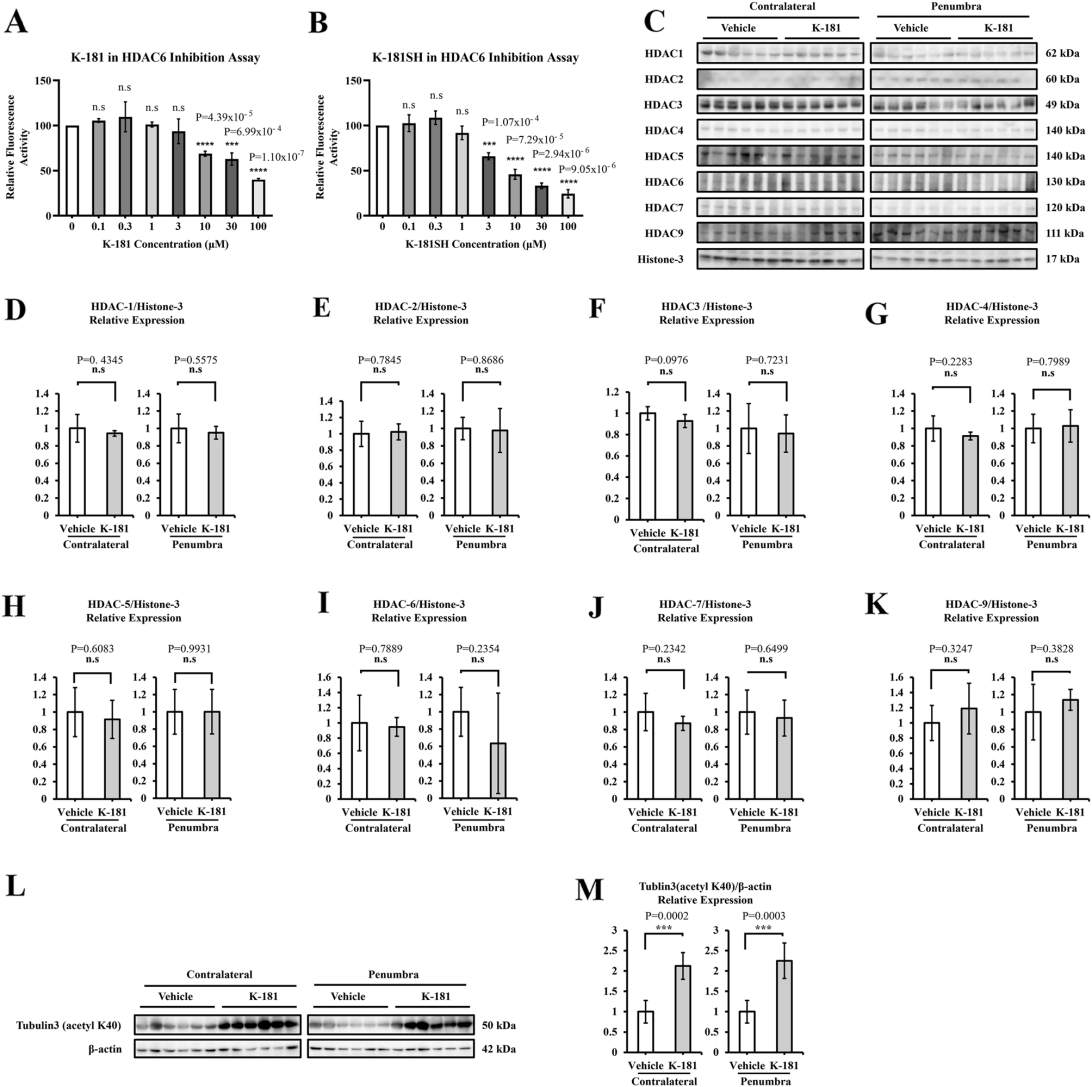
K-181 selectively inhibits HDAC6 activity and enhances Tubulin acetylation. (**A**,**B**) HDAC6 inhibition assay of 0.1, 0.3, 1, 3, 10, 30, 100 μM K-181 (**A**) and K-181SH (**B**). N = 3. (**C**–**K**) Western Blot images (**C**) and quantification of HDAC1 (**D**), HDAC2 (**E**), HDAC3 (**F**), HDAC4 (**G**), HDAC5 (**H**), HDAC6 (**I**), HDAC7 (**J**) and HDAC9 (**K**) expression in vehicle and K-181 treated mice brains. N = 6. (**L**,**M**) Western Blot images (**L**) and quantification (**M**) of Tubulin3 (acetyl K40) in vehicle and K-181 treated mice brains. N = 6.

Our group have so far synthesized multiple Histone Deacetylases (HDACs) inhibitors^47,50,51^. HDACs are grouped into 4 classes and classified as 18 isoforms. We evaluated HDAC activity inhibition of K-181, K-178 and their free thiol derivatives. A pan-HDAC inhibitor Trichostatin A (TSA), and a typical HDAC6 inhibitor Tubacin, were adopted as positive controls. As HDAC enzymes, HDAC1, HDAC4 and HDAC6 were used as representative HDAC class I (HDAC1/2/3/8), class IIa (HDAC4/5/7/9) and class IIb (HDAC6/10), respectively. HDAC enzyme activities were recorded and only compounds that inhibition (%) > 50 were defined to have an inhibitory effect. Compared to TSA, K-181 showed a selective inhibitory effect on HDAC6 activity but barely influenced HDAC1 and HDAC4 activity (Table 1). K-181 showed a dose-dependent inhibitory effect on HDAC6 activity from 10 to 100 μM (Fig. 6A). K-181SH, an active form of K-181 in vivo, showed a significant HDAC6 activity inhibition at 3 μM (Fig. 6B). We detected the main HDAC isoforms protein expression in mice brain, from both in penumbra and contralateral to ischemia (Fig. 6C). K-181 orally treated mice showed no statistically significant inhibitory effects on HDAC-1 (Fig. 6D), HDAC2 (Fig. 6E), HDAC3 (Fig. 6F), HDAC4 (Fig. 6G), HDAC5 (Fig. 6H), HDAC6 (Fig. 6I), HDAC7 (Fig. 6J) and HDAC9 expression (Fig. 6K). Tubulin acetylation is crucial for cell stability, especially when exposed to exotic damage like ischemia/reperfusion and could be regulated by HDAC6. K-181 treated mice displayed an enhanced acetylation of Tubulin-3, under both physiological and ischemic conditions (Fig. 6L,M).

**Table 1:** HDAC1, HDAC4 and HDAC6 inhibition assay of K-178, K-178SH, K-181, K-181SH, Tubacin and Trichostatin A.

Compound tested	HDAC1 activity assay				HDAC4 activity assay				HDAC6 activity assay			
	Inhibition (%)		Average of Inhibition (%)	Inhibitory activity*	Inhibition (%)		Average of Inhibition (%)	Inhibitory activity*	Inhibition (%)		Average of Inhibition (%)	Inhibitory activity*
K-178	17.75	7.93	12.84	N/A	13.19	6.95	10.07	N/A	39.74	30.59	35.16	N/A
K-178SH	5.56	12.23	8.9	N/A	9.89	8.1	8.99	N/A	41.16	35.49	38.32	N/A
K-181	16.76	17.1	16.93	N/A	12.22	12.22	12.22	N/A	55.51	59.12	57.32	Yes
K-181SH	21.58	24.73	23.15	N/A	14.38	16.3	15.34	N/A	68.85	70.78	69.81	Yes
Tubacin	97.65	97.22	97.43	Yes	87.71	87.27	87.49	Yes	100	100	100	Yes
Trichostatin A	99.94	99.61	99.78	Yes	97.7	97.51	97.61	Yes	99.37	99.54	99.45	Yes

*HDAC inhibitory effect is defined as “Average of Inhibition (%)” > 50.

**Figure 7:**
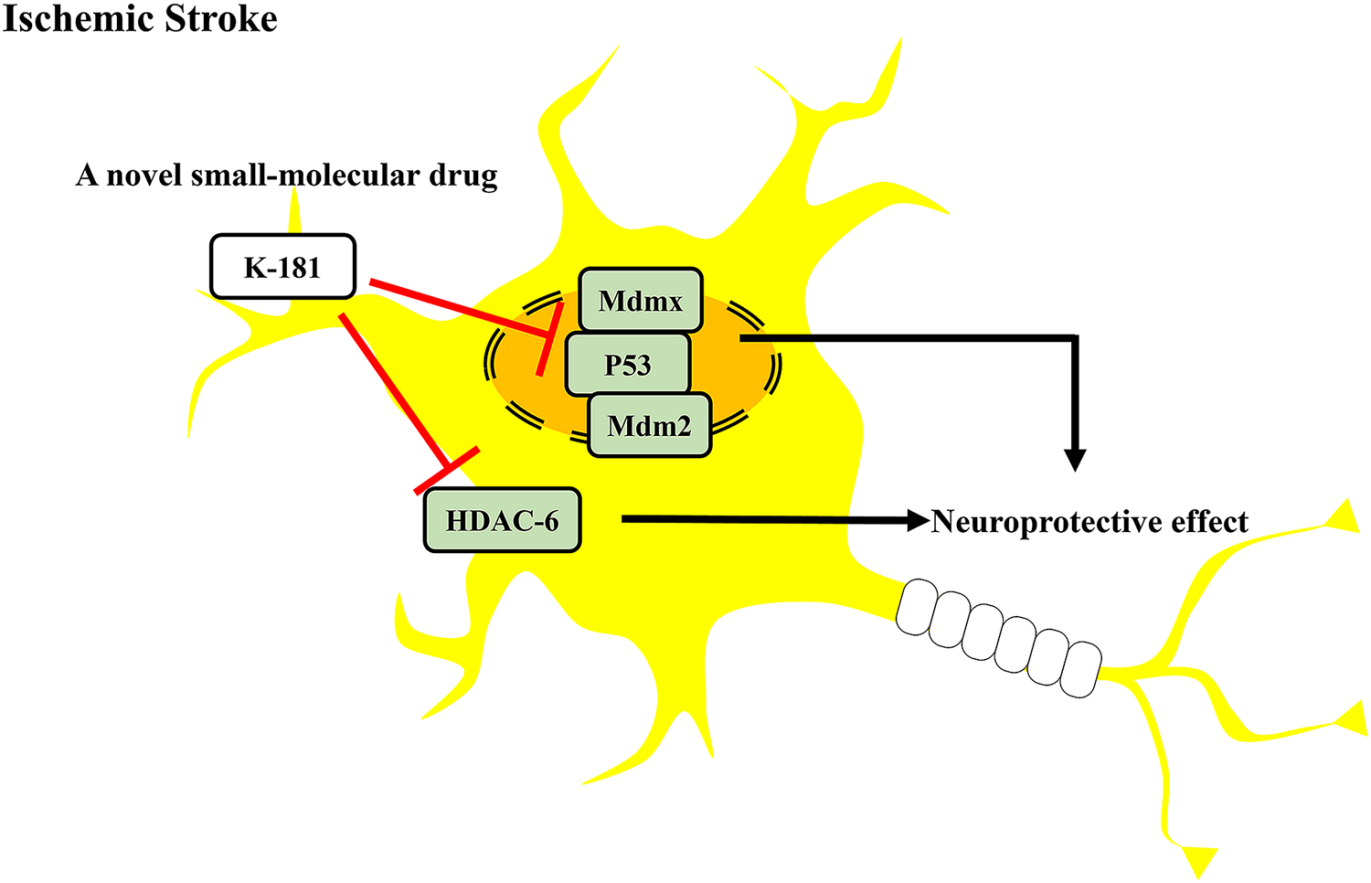
K-181 exerts neuroprotective effects by modifying the interactions between Mdmx and p53 and selectively inhibiting HDAC6 activity.

Taken together, these results suggest K-181 and its derivatives have neuroprotective effects on ischemic stroke by disrupting Mdmx–p53 interaction and inhibiting HDAC6 activity (Fig. 7). This may provide a novel approach for ischemic stroke therapy.

## Discussion

Many PPI inhibitors of interaction between p53 and its binding proteins have been developed in cancer. Despite the crucial role of p53 for cell death in neurological disorders, few reports have been made on the development of neuroprotective treatments with PPI inhibitors of Mdmx–p53 and Mdm2–p53. K-181, our newly developed small-molecule Mdmx–p53 inhibitor, reduced neuronal damage and attenuated the neurological deficits after stroke by increasing the expression of Mdmx protein.

Somatic p53 mutations are found in approximately 50% of human cancers^52,53^. The function of p53 is impaired either by the mutations or multiple upstream negative regulators including in Mdm2 and Mdmx. Although many phase I, II and III clinical trials with small molecule compounds that reactivate p53 are currently underway, there remains certain limitations and concerns. Unlike its mutations difficult to target in cancers, p53 is often wild-type in mature neurons which are impaired by stroke and neurodegenerative diseases, and the discovery of therapeutic drugs targeting Mdm2/Mdmx–p53 interactions in clinics can be potentially useful in AML, multiple myeloma, and other hematological malignancies^54^.

Multiple drugs have been synthesized to target Mdm2–p53 interaction up to now^33,34^. Nutlin-3a, a well-known Mdm2–p53 inhibitor with a molecular weight of 581, led to an increased vulnerability to ischemic neuronal injury, which could be reversed by p53 Knock-out. Furthermore, Patients harboring G allele in Mdm2 promoter (Single-Nucleotide Polymorphism 309 T>G) were reported to have a higher Mdm2 protein levels and better functional outcomes after stroke than those harboring the T/T genotype^55^.

Researches on Mdmx–p53 interaction inhibitor remain few. The first small-molecule Mdmx inhibitor is SJ-172550. However, we did not find a neuroprotective effect on ischemic neuronal damage by administration of SJ-172550 in this work. The novel small-molecular weight drug, K-181 preferentially disrupted Mdmx–p53 interaction over Mdm2–p53 interaction in mice brain and showed high binding affinity to Mdmx in vitro. Furthermore, K-181 oral administration increased Mdmx expression in vivo by a dose-dependent way, and exerted an inhibitory effect on p53, Mdm2 and E2F1 expression. In addition, K-181 showed a neuroprotective effect in acute ischemic stroke, which is supported by the downregulation of apoptotic regulators like cleaved Caspase-3 and proinflammatory factors like TNF-α.

The neuroprotective effect of Mdmx may be both p53-dependent and p53-independent mechanism. Mdmx is phosphorylated at multiple sites in response to cell stresses, and these modifications play critical roles in different signaling pathways, especially in apoptosis and necrosis^56^. Previous studies have reported that Mdmx interacts with other apoptosis modulating proteins, such as E2F1^57^, suggesting that Mdmx may play a critical role against neuronal death. Consistent with these reports, our study demonstrated that K-181 ameliorates mice neuronal death, accompanied with a decrease in E2F1 protein. Additionally, Mdmx has recently been reported to be p53-independently associated with CK1α activity^58^, DNA replication^59^, and ferroptosis^60^. Further investigation is needed on the involvement of these p53-independent mechanisms of Mdmx in cerebral ischemia.

Interestingly, K-181 also had an inhibitory effect on HDAC6 activity by oral administration and restored tubulin acetylation. HDAC6 is involved in tau aggregation and phosphorylation in neurons and human AD brain^61,62^, and Ricolinostat (ACY-1215) is the first oral selective HDAC6 inhibitor with reduced class I HDAC activity to be studied clinically for treatment of multiple myeloma^63^. Moreover, HDAC6 directly deacetylates Lys120^64^ and Lys381/382^65^ of p53 to coordinate p53-dependent signaling pathway, which further explains the neuroprotective effects of K-181 despite the p53-independent neuroprotection of K-181 induced Mdmx increase. The clarification of p53-dependent and p53-independent mechanisms of K-181 and Mdmx deserves further investigation.

At present, most of PPI inhibitors are medium and large weight molecules, typified by peptides and antibodies. In this work, we discovered a small molecule PPI inhibitor K-181, and showed a neuroprotective effect of K-181 that disrupts Mdmx–p53 interaction and enhances Mdmx expression. Further studies are needed to clarify the underlying mechanism of its inhibitory regulation on Mdmx/Mdm2–p53 axis. This study may offer a novel insight into the future ischemic stroke therapy.

## Supplementary Information

Supplementary Figures.

Supplementary Information.

## Data Availability

The data generated during the current study are available from the corresponding author on reasonable request. All the original blots used in figures were included in Supplementary Figs. 5–10.
