# Supplementary material for: MDMX elevation by a novel Mdmx–p53 interaction inhibitor mitigates neuronal damage after ischemic stroke
Source: Sci Rep. 2022 Dec 6;12:21110. doi: 10.1038/s41598-022-25427-4 (PMC9726886; doi:10.1038/s41598-022-25427-4)
Supplement: Supplementary file 1 — Supplementary Figures. [file 41598_2022_25427_MOESM1_ESM.docx]

**Supplemental Figures**

**MDMX elevation by a Novel Mdmx-p53 interaction Inhibitor Mitigates Neuronal Damage after Ischemic Stroke**

Haomin Yan^1^, Tsutomu Sasaki^1,2^*, Hideaki Kanki^1^, Yoshiyuki Hirata^3^, Kumiko Nishiyama^1^, Sunao Hisada^4^, Shigenobu Matsumura^5^, Yasuo Nagaoka^6^, Takaaki Sumiyoshi^6^, Seiichi Nagano^1,2^, Akiko Nakata^7^, Minoru Yoshida^7,8^, Shinichi Uesato^3^, Hideki Mochizuki^1^

^1^Department of Neurology, Graduate School of Medicine, Osaka University, Yamadaoka 2-2, Suita, Osaka, 565-0871, Japan.

^2^Department of Neurotherapeutics, Graduate School of Medicine, Osaka University, Yamadaoka 2-2, Suita, Osaka, 565-0871, Japan.

^3^Faculty of Pharmacy, Osaka Medical and Pharmaceutical University, 4-20-1 Nasahara, Takatsuki, Osaka 569-1094, Japan.

^4^Hamamatsu Photonics, K.K. System Division, Joko-cho, Hamamatsu, Shizuoka 431-3196, Japan

^5^Graduate School of Comprehensive Rehabilitation, Osaka Prefecture University,

Osaka, 583-8555, Japan

^6^Department of Life Science and Biotechnology, Faculty of Chemistry, Materials and Bioengineering, Kansai University, Yamate-cho 3-3-35, Suita, Osaka 564-8680, Japan

^7^Seed Compounds Exploratory Unit for Drug Discovery Platform, RIKEN Center for Sustainable Resource Science, 2-1 Hirosawa, Wako, Saitama 351-0198, Japan

^8^Chemical Genomics Research Group, RIKEN Center for Sustainable Resource Science, 2-1 Hirosawa, Wako, Saitama 351-0198, Japan

*Correspondence to: Tsutomu Sasaki

Department of Neurology, Graduate School of Medicine, Osaka University, Yamadaoka 2-2, Suita, Osaka 565-0871, Japan

Fax: +81-6-6879-3579; Tel: +81-6-6879-3571

E-mail: sasaki@neurol.med.osaka-u.ac.jp


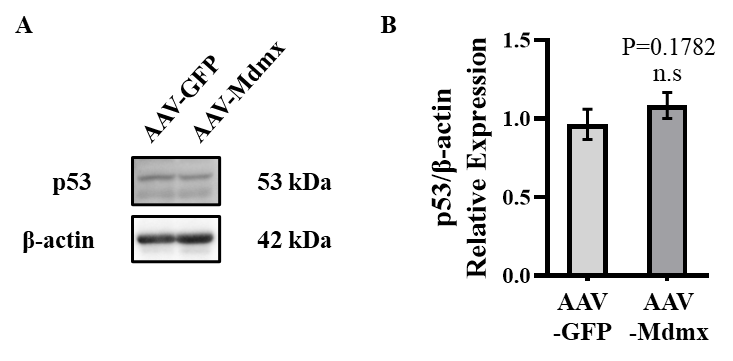


**Supplemental Figure 1. Western blot images (A) and quantification (B) of p53 in neurons transfected with AAV-GFP and AAV-Mdmx.** N = 3.


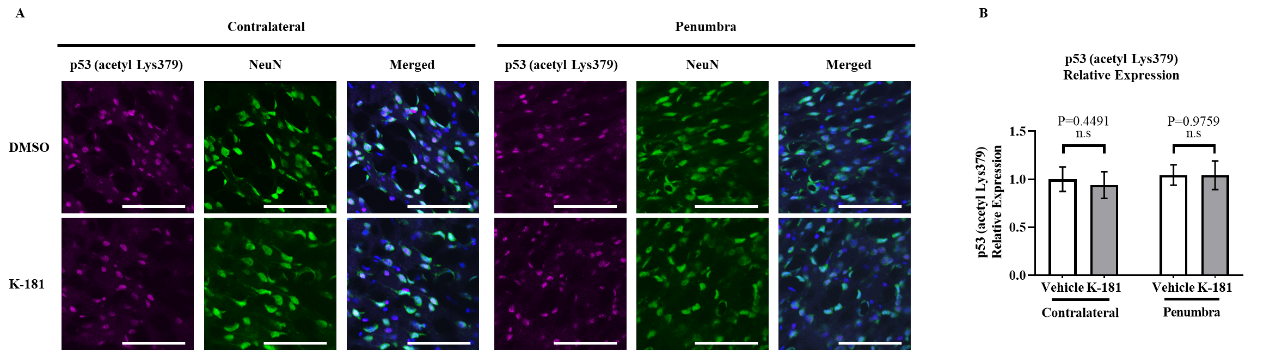


**Supplemental Figure 2.** **Representative immunostaining images (A) and quantification of p53 (acetyl Lys379) (B) in vehicle and K-181 treated mice brains.** N = 6. Scale Bar = 100 μm.


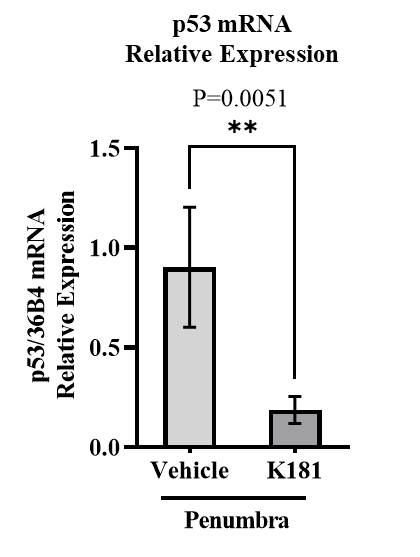


**Supplemental Figure 3. qPCR of p53 mRNA levels in vehicle and K-181 treated mice penumbra.** N = 3.


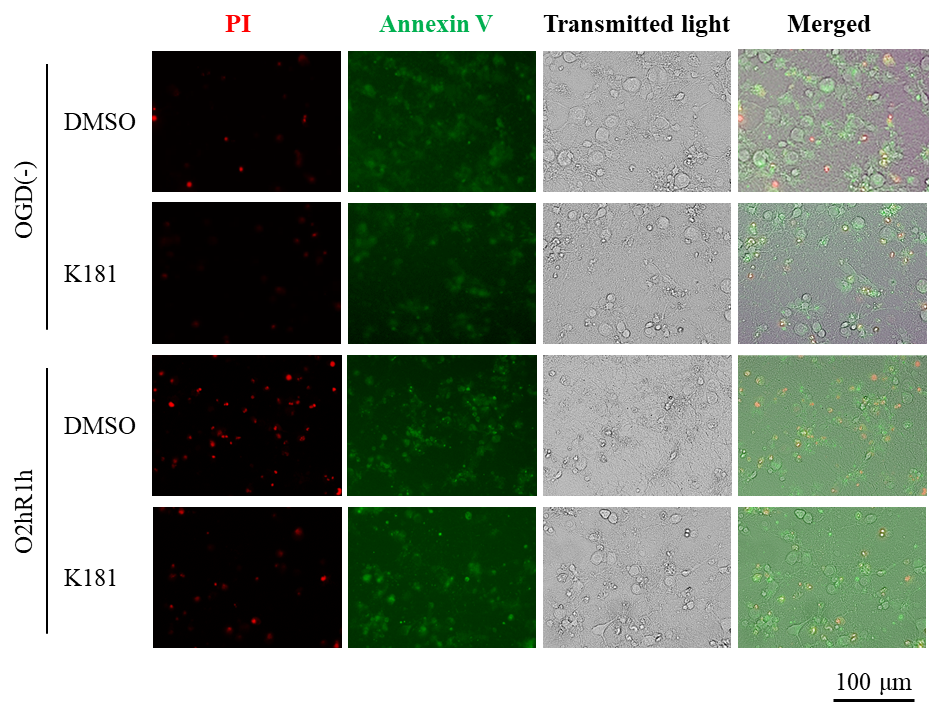


**Supplemental Figure 4.** Annexin V translocation assay of neurons treated with DMSO or K-181, before and after OGD/reperfusion.


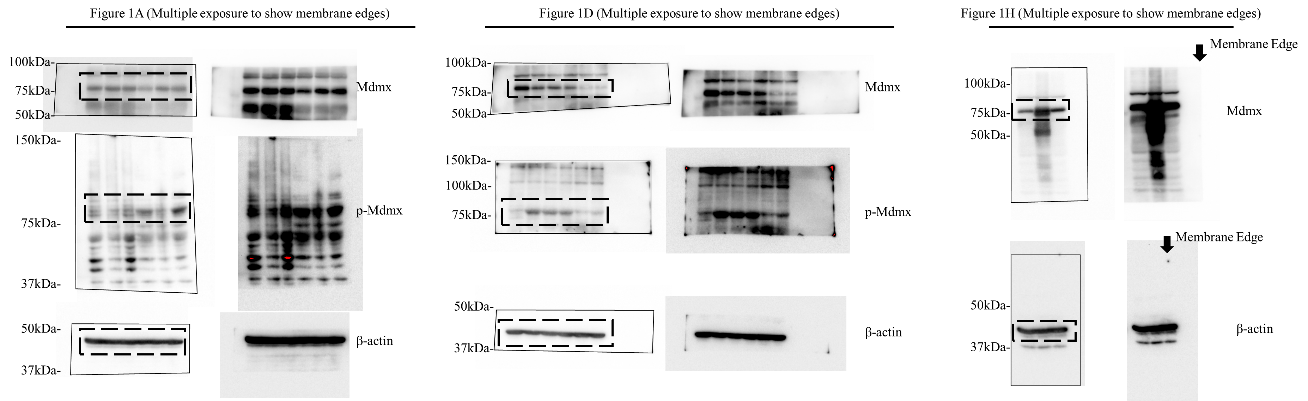


**Supplemental Figure 5.** **Original western blot images reported in Figure 1.** The original western blot images reported in Figure 1A (for Mdmx, p-Mdmx and β-actin), in Figure 1D (for Mdmx, p-Mdmx and β-actin) and in Figure 1H (for Mdmx and β-actin) have been highlighted with box.

**
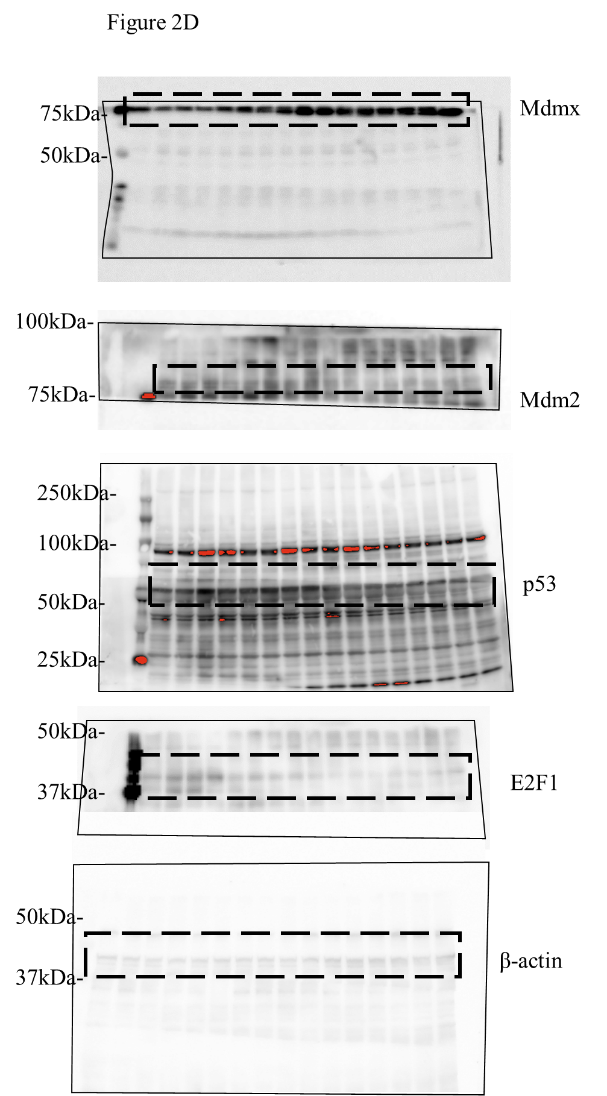
**

**Supplemental Figure 6.** **Original western blot images reported in Figure 2D.** The original western blot images reported in Figure 2D (for Mdmx, Mdm2, p53, E2F1 and β-actin) have been highlighted with box.

**
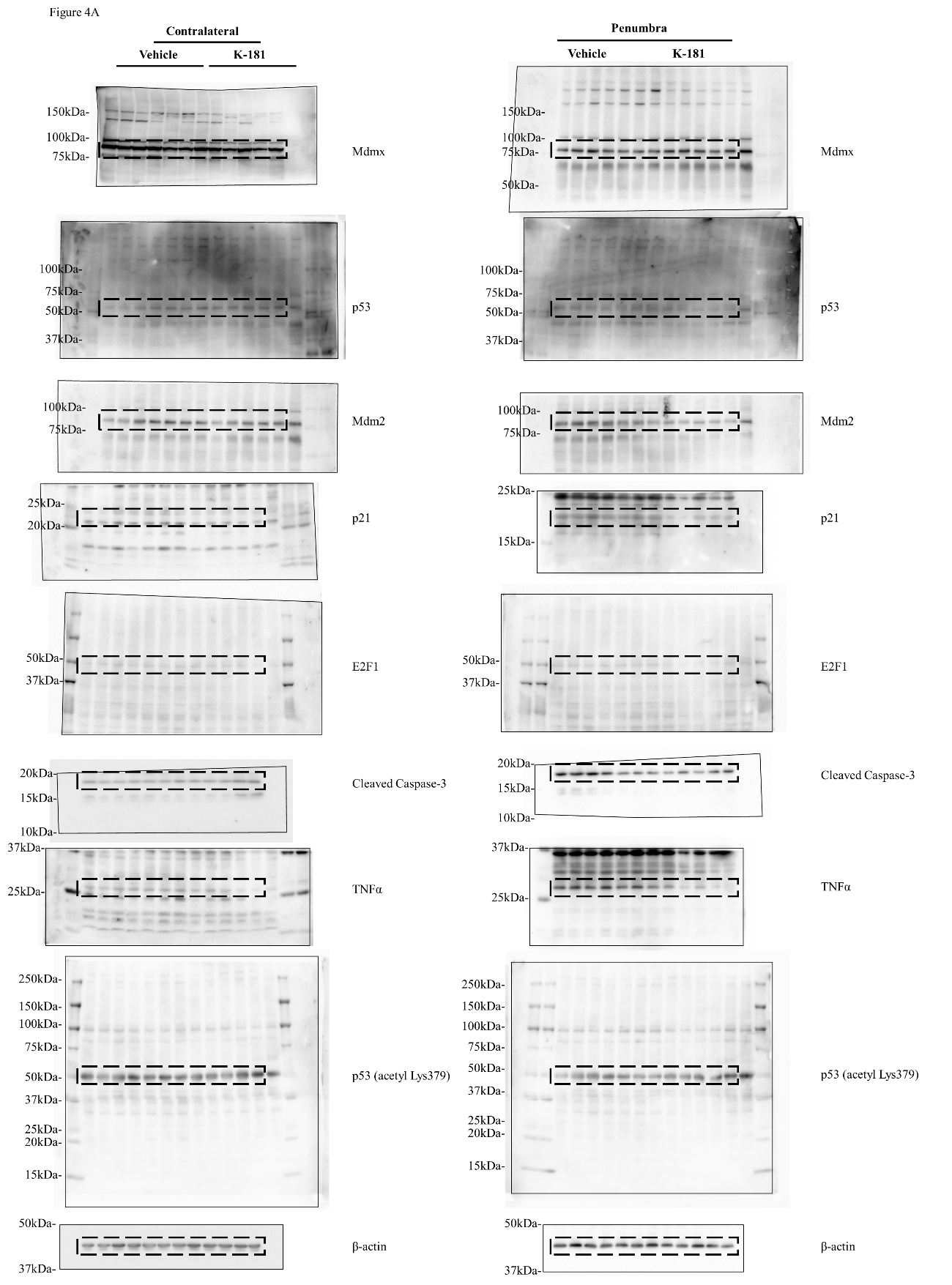
**

**Supplemental Figure 7.** **Original western blot images reported in Figure 4A.** The original western blot images reported in Figure 4A (for Mdmx, p53, Mdm2, p21, E2F1, cleaved Caspase-3, TNFα, p53 (acetyl Lys379) and β-actin) have been highlighted with box.

**
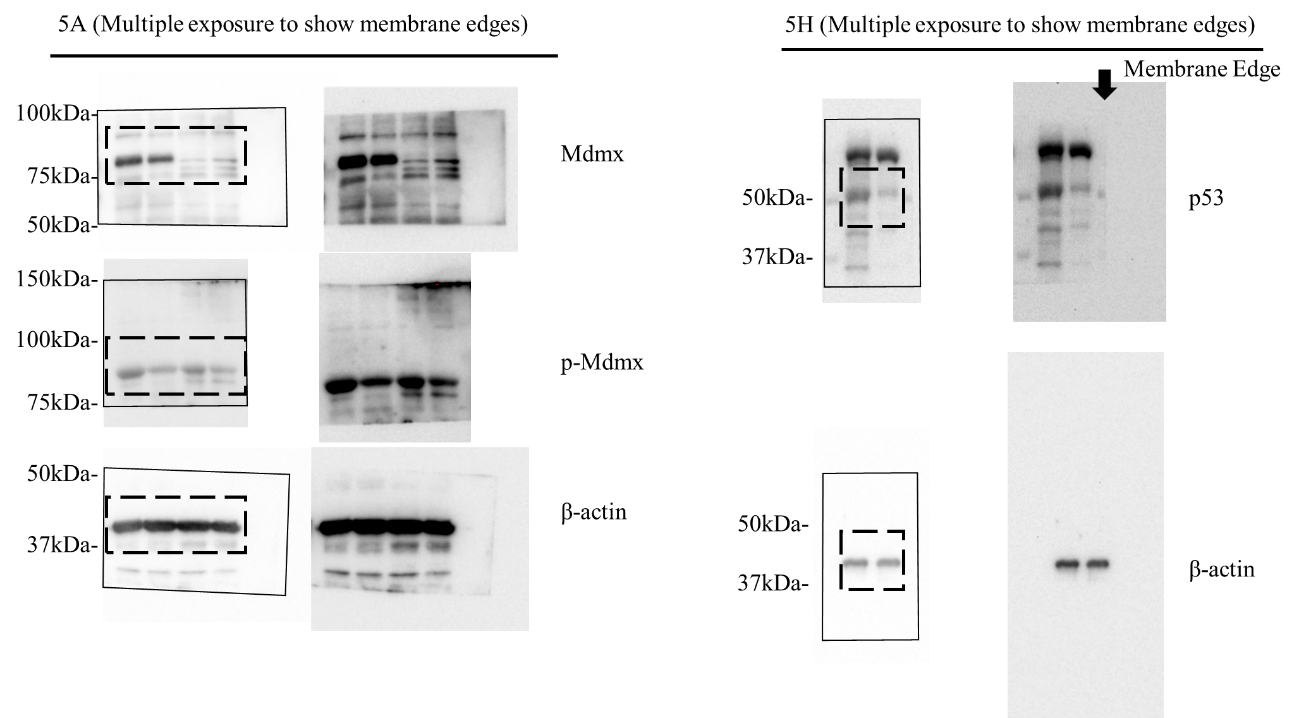
**

**Supplemental Figure 8.** **Original western blot images reported in Figure 5.** The original western blot images reported in Figure 5A (for Mdmx, p-Mdmx and β-actin) and in Figure 5H (for p53 and β-actin) have been highlighted with box.

**
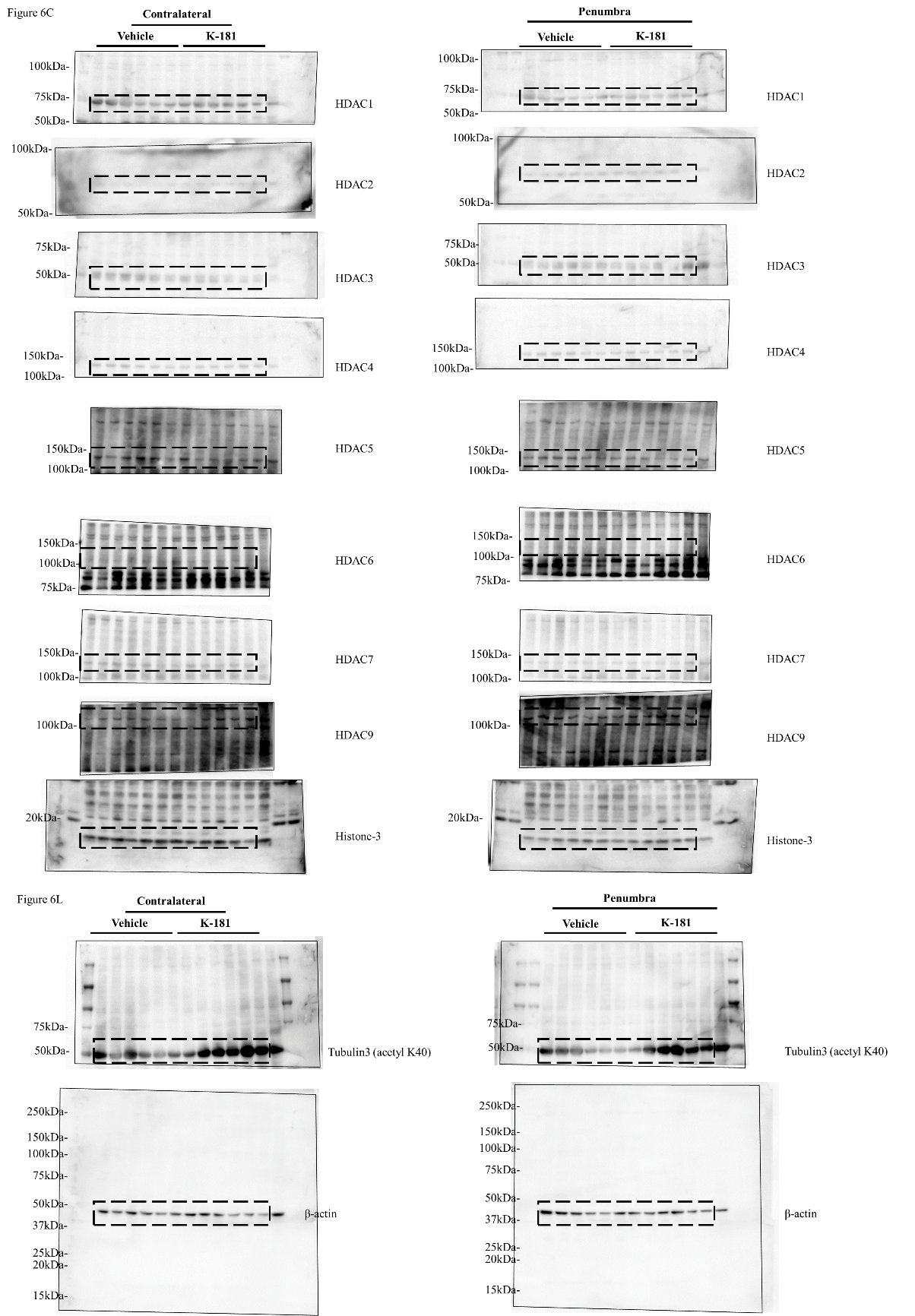
**

**Supplemental Figure 9.** **Original western blot images reported in Figure 6.** The original western blot images reported in Figure 6C (for HDAC1, HDAC2, HDAC3, HDAC4, HDAC5, HDAC6, HDAC7, HDAC9 and Histone-3) and in Figure 6L (for Tubulin3 (acetyl K40) and β-actin) have been highlighted with box.

**
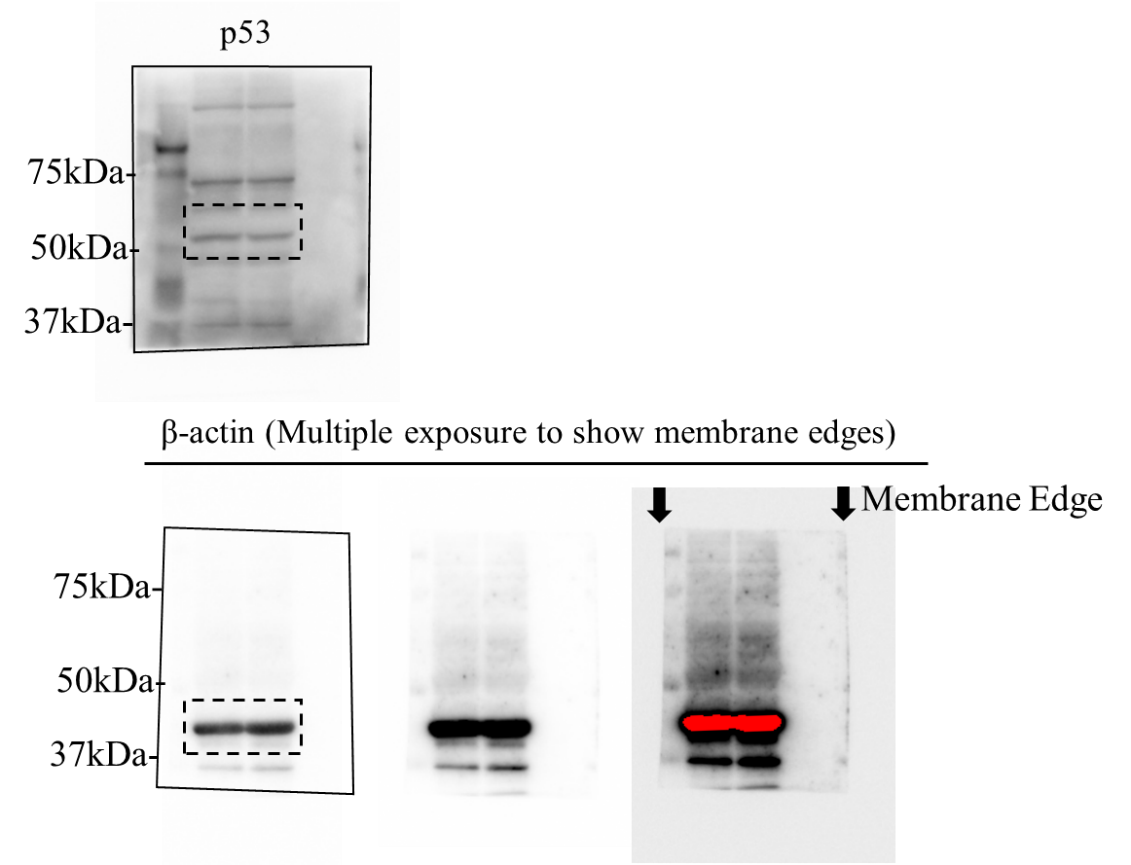
**

**Supplemental Figure 10.** **Original western blot images reported in Supplemental Figure 1.** The original western blot images reported in Figure Supplemental Figure 1 (for p53 and β-actin).
