# Supplementary material for: MDMX elevation by a novel Mdmx–p53 interaction inhibitor mitigates neuronal damage after ischemic stroke
Source: Sci Rep. 2022 Dec 6;12:21110. doi: 10.1038/s41598-022-25427-4 (PMC9726886; doi:10.1038/s41598-022-25427-4)
Supplement: Supplementary file 2 — Supplementary Information. [file 41598_2022_25427_MOESM2_ESM.docx]

**Supplemental Methods**

**p53-mdmx/mdm2 interaction inhibition assay**

p53-mdmx interaction was assessed by the LANCE TR-FRET (Lanthanide Chelate Excite time-resolved fluorescence resonance energy transfer) system(1). GST-mdmx was prepared according to a previous report(2). p53-His6 constructs in Gateway™ pDEST™ 17 Vectors (Thermo Scientific) were transformed into Escherichia coli BL21 (DE3) (Novagen) by the heat shock at 40 ℃ for 30 sec. Cells were cultured at 37 ℃ on LB agar medium containing 100 μg/mL ampicillin. The cells were cultured in LB liquid medium containing 100 μg/mL ampicillin at 37 ℃ until OD600 reached 0.5. The culture solution was induced by 0.1 mM IPTG (Nacalai Tesque) at 25 ℃ for 3 h and was centrifuged at 8000 rpm for 20 min. The pellets were collected, resuspended in lysis buffer (1×PBS containing 0.1 % Tween 20, 0.05% DTT, and 0.05 % PMSF), and sonicated on ice. The lysates were centrifuged at 8000 rpm for 20 min, and were purified by HisTrapTM HP column (GE healthcare) and Sephadex G-75 column (GE healthcare) using the AKTA Explorer system (GE healthcare). p53-His6 (80 nM, 2.5 μL) and GST-mdmx (200 nM, 2.5 μL) and test compounds (50 μM, 5 μL) add to 1/2 AreaPlateTM-96 (Perkin Elmer) and the mixture reacted at room temperature for 1 h. The reacted mixture was added to the working solution, which the europium donor, Anti-6X-His Eu W1024 (5 μL) (Perkin Elmer) and the europium acceptor, Anti-GST ULight (5 μL) (Perkin Elmer). The assay plate was incubated at room temperature for 1 h in the dark, and measured by EnSpire Multimode Plate Reader (Perkin Elmer).

**2,3,5-Triphenyltetrazolium chloride (TTC) staining**

After 1 day of reperfusion, mice were sacrificed and brains were sectioned at 2 mm intervals, stained with 2% TTC at room temperature for 30 min. Brain sections were photographed with a digital microscope (Olympus, SZX12). The infarct volume was calculated as: whole contralateral hemisphere volume − nonischemic ipsilateral hemisphere volume.

**Primary neuronal cultures**

Briefly, neuronal cultures were prepared from the cortex of embryonic day 16 (E16) mouse embryos. Cortical tissue was incubated in dissection medium (Dulbecco’s modified Eagle’s medium containing 100 U of papain and 0.5 mg/ml DNase type II) for 30 min at 37 °C. After centrifugation, cells were plated onto 6 well plates and 100-mm dishes (Corning, NY, USA) coated with polyethylenimine. Cells were cultured to a final concentration of 7.0 × 10^5^ cells/ml in high-glucose DMEM (Wako, Osaka, Japan) containing 10% fetal calf serum (Invitrogen) and 100 IU/ml penicillin. After 24 h, the medium was changed to Neurobasal medium (Invitrogen) supplemented with B-27 (Invitrogen).

**Oxygen Glucose Deprivation (OGD)**

Cultures were washed with phosphate-buffered saline (PBS) and incubated in glucose-free Earle’s balanced salt solution (EBSS) (Biological Industries, Israel) under an anaerobic environment of 95% N_2_/5% CO_2_, maintaining an O_2_ pressure of 10–15 Torr, at 37 °C for 3.5 h. After incubation, OGD was terminated and cultures were returned to the normoxic chamber.

**Cell viability Assay**

The Cytotoxicity Detection Kit (Roche) was used to evaluate neuronal death. The cytotoxicity detection buffer was mixed and added into collected supernatant according to the manufacturer’s instructions. After 30 min incubation at room temperature, the OD of each mixture was measured with an absorbance microplate reader (CORONA SH-9000Lab) at 490 and 650 nm. Neuronal cytotoxicity was calculated as: LDH release (OD_490_) / LDH release (OD_650_). 100% cell death rate was revised to 2% Triton X-100 (Wako) pre-added in neuronal cultures before supernatant collection. Background control was recorded as absorbance value of 100 μl cytotoxicity detection buffer mixture. Neuron death rate % was calculated as (Example value – Background control) / (Maximum LDH release – Background control) .

**Annexin V assay**

Annexin V translocation was measured before and 1 h after 2 h OGD using Annexin V-FITC Apoptosis Detection Kit (Nacalai). Briefly, cultures were washed with cold PBS, then incubated 15 min at room temperature with Annexin V-FITC conjugate and propidium iodide (PI) (1:1000 dilution in Annexin V Binding Soution). Samples were imaged on Keyence 810 microscopy.

**Western blotting**

Brains and cultures were collected and lysed in TNE buffer on ice. Protein content was quantified with the BCA Protein Assay Kit (Thermo Fisher Scientific). 10 μg protein samples were electrophoresed and transferred to PVDF membranes (Merck Millipore, Burlington, MA, United States). After 1 h blocking with 5% non-fat dry milk, membranes were incubated with primary antibody overnight at 4 °C. After 1 h of secondary antibody incubation at room temperature, visualization was conducted with a luminoimage analyzer (Chemidoc Touch, Bio-Rad Laboratories, CA, USA). The following antibodies were used: Mdmx (1:1000; Proteintech, Rosemont, IL, USA); p-Mdmx (1:1000; Bioacademia, Osaka, Japan); Mdm2 (1:1000; Sigma-Aldrich, St. Louis, MO, USA); p53 (1:1000; Sigma-Aldrich); p21 (1:1000; Cell Signaling Technology, Danvers, MA, USA); E2F1 (1:1000; Cell Signaling Technology); Cleaved Caspase-3 (1:1000; Cell Signaling Technology); TNF-α (1:1000; Cell Signaling Technology); β-actin (1:5000; Proteintech, Rosemont); HDAC-1 (1:1000; Cell Signaling Technology); HDAC-2 (1:1000; Cell Signaling Technology); HDAC-3 (1:1000; Cell Signaling Technology); HDAC-4 (1:1000; Cell Signaling Technology); HDAC-5 (1:1000; Cell Signaling Technology); HDAC6 (1:1000; Abcam, Cambridge, UK); HDAC-7 (1:1000; Cell Signaling Technology); HDAC-9 (1:1000; Abcam); Histone-3 (1:1000; Cell Signaling Technology); Tubulin-3(Acetyl K49) (1:1000; Sigma-Aldrich); Tubulin (1:1000; Sigma-Aldrich).

**Immunofluorescence Staining**

For immunofluorescence staining *in vitro*, neurons were cultured on slides. After fixation with 4% PFA at room temperature for 30 min, slides were washed 3 times with PBS and then incubated with 0.3% TritonX-100 at room temperature for 30 min. After 30 min of blocking with 10% donkey serum in TBS, slides were incubated with MAP2 (1:500; Invitrogen), Mdmx (1:250; proteintech) and phosphorylated CREB (1:250; Millipore) antibodies. Alexa Fluor 350-labeled donkey anti-rabbit IgG, 647-labeled donkey anti-mouse IgG, 488-labeled donkey anti-mouse IgG and 647-labeled donkey anti-rabbit IgG (Invitrogen) were used as secondary antibody.

For immunofluorescence staining *in vivo*, Mice were sacrificed and perfused with iced PBS, followed by 2% paraformaldehyde. Brains were isolated and postfixed in 2% paraformaldehyde for 24 h and gradually dehydrated, then cut into 14 μm - thick sections. Sections were incubated with 0.1% TritonX-100 at room temperature for 30 min, then incubated with 10% donkey serum in PBS. Slides were incubated with primary antibodies overnight at 4 °C, then subjected to 3 h of secondary antibody incubation at room temperature. Brain sections were counterstained with DAPI (Vector Laboratories), and a confocal laser-scanning microscope (Olympus FV3000) was used for visualization to avoid interference between channels and saturation. The following antibodies were used: p53 (1:250; Santa Cruz Biotechnology), p53 (Acetyl Lys379) (1:250; Sigma Aldrich), NeuN (1:250,Chemicon), Caspase-3 (1:250; Cell Signaling Technology). Quantification of immunofluorescence staining was conducted on 6 images which were obtained using 20x objective from 3 independent slides.

**References**

1. Hemmila II. LANCEtrade mark: Homogeneous Assay Platform for HTS. J Biomol Screen. 1999;4(6):303-8.

2. Uesato S, Matsuura Y, Matsue S, Sumiyoshi T, Hirata Y, Takemoto S, et al. Discovery of new low-molecular-weight p53-Mdmx disruptors and their anti-cancer activities. Bioorg Med Chem. 2016;24(8):1919-26.
